# Variation in genome size, cell and nucleus volume, chromosome number and rDNA loci among duckweeds

**DOI:** 10.1038/s41598-019-39332-w

**Published:** 2019-03-01

**Authors:** Phuong T. N. Hoang, Veit Schubert, Armin Meister, Jörg Fuchs, Ingo Schubert

**Affiliations:** 1 Leibniz Institute of Plant Genetics and Crop Plant Research (IPK) Gatersleben, D-06466 Seeland, Germany; 2 Present Address: Dalat University, Lamdong Province, Vietnam

## Abstract

Duckweeds are small, free-floating, largely asexual and highly neotenous organisms. They display the most rapid growth among flowering plants and are of growing interest in aquaculture and genome biology. Genomic and chromosomal data are still rare. Applying flow-cytometric genome size measurement, microscopic determination of frond, cell and nucleus morphology, as well as fluorescence *in situ* hybridization (FISH) for localization of ribosomal DNA (rDNA), we compared eleven species, representative for the five duckweed genera to search for potential correlations between genome size, cell and nuclei volume, simplified body architecture (neoteny), chromosome numbers and rDNA loci. We found a ~14-fold genome size variation (from 160 to 2203 Mbp), considerable differences in frond size and shape, highly variable guard cell and nucleus size, chromosome number (from 2n = 36 to 82) and number of 5S and 45S rDNA loci. In general, genome size is positively correlated with guard cell and nucleus volume (p < 0.001) and with the neoteny level and inversely with the frond size. In individual cases these correlations could be blurred for instance by particular body and cell structures which seem to be linked to specific floating styles. Chromosome number and rDNA loci variation between the tested species was independent of the genome size. We could not confirm previously reported intraspecific variation of chromosome numbers between individual clones of the genera *Spirodela* and *Landoltia*.

## Introduction

Duckweeds comprise 37 species within 5 genera: *Spirodela* (2 species), *Landoltia* (1), *Lemna* (13), *Wolffiella* (10) and *Wolffia* (11)^1,2^. All duckweeds are lacking the morphological differentiation of seed plants into stems, branches and leaves, and from *Spirodela* toward *Wolffiella* and *Wolffia* the roots are gradually lost too. This morphological reduction is called neoteny^3^ in analogy to animals which maintain embryonic features as adults. Duckweeds are small, free-floating, aquatic plants. They belong to the monocot order *Alismatales* and display highly reduced organs and the fastest growth rate among flowering plants. The leaf-like organism structure of duckweeds which lacks a stem is called “frond”. In the phylogenetically youngest genera *Wolffiella* and *Wolffia* even roots are lacking. Although (at least occasionally) flowers were observed in most species, e.g. in *Wo*. *microscopica*^4^, *Wo*. *australiana*^5^ and *Wo*. *arrhiza*^6^, duckweeds usually reproduce asexually by forming daughter fronds from meristematic pockets (primordia) at the proximal end of a mother frond^3,7,8^.

Two Lemnaceae monographs of Elias Landolt provide fundamental insights into biodiversity, morphology, ecology, physiology and the development of duckweeds^9,10^.

Genome size can be a diagnostic feature of individual species and contributes to the elucidation of whole genome duplication (WGD) and other events during genome evolution. During the last decades, flow-cytometry became the preferred method for genome size measurement in plants. Besides the easiness of sample preparation and high throughput, the capability to estimate genome size, nuclear replication state, ploidy and endopolyploidy levels are advanced features of this method compared to other approaches such as Feulgen densitometry or genome sequencing^11^. The genome size has been established for different duckweed species. No significant differences were detected between the genome sizes of the two *Spirodela* species *S*. *polyrhiza* and *S*. *intermedia* (both 160 Mbp). The genus *Landoltia* comprises only one species *(La*. *punctata)* with a genome size of 421 Mbp^12^. A correlation between genome size evolution, frond size and neoteny level was observed by Wang *et al*.^13^ when investigating 115 clones of 23 out of 37 duckweeds species. For some individual species Wang *et al*.^13^ and Bog *et al*.^12^ reported different genome sizes. These differences might be due to the use of different internal reference standards, to true differences between clones, or simply to random variation between measurements.

Interestingly, duckweed frond sizes vary from 1.5 cm to less than 1 mm in diameter accompanied by a nearly 12-fold genome size variation (from 160 Mbp to 1881 Mbp according to Wang *et al*.^13^) and a successive reduction of morphological structures from *Spirodela* towards *Wolffia* species^9,12,13^. This potential correlation of genome size with morphological reduction and frond size evolution makes duckweeds an interesting subject for genome and karyotype evolution studies. A positive correlation between nuclear DNA content and nuclear and cell volume was recorded for some angiosperms^14^ and for endosperm cells of *Sorghum bicolor*^15^. To elucidate whether also for duckweeds a correlation between genome size, cell and nuclear volume is valid, accessions of eleven representative species of the five duckweed genera were investigated. Additionally, we studied the chromosome number and genomic distribution of 5S and 45S rDNA loci of these species.

## Results

### Differences in morphology between duckweed genera

**Figure 1:**
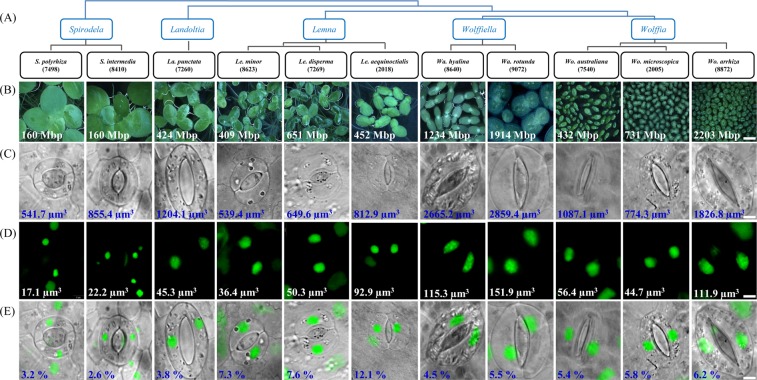
Phylogenetic relationship, frond, stomata and nuclei morphology of duckweed species. (**A**) Phylogenetical position. (**B**, **C**) Differences in size and morphology of fronds and stomata. (**D**,** E**) Nuclei shape and distribution within the guard cells. Numbers indicate genome size (**B**), average cell (**C**) and nuclear volumes (**D**), and percentage of nuclear to cell volume (**E**). Scale bars = 200 µm (**B**) and 5 µm (**C**–**E**).

**Figure 2:**
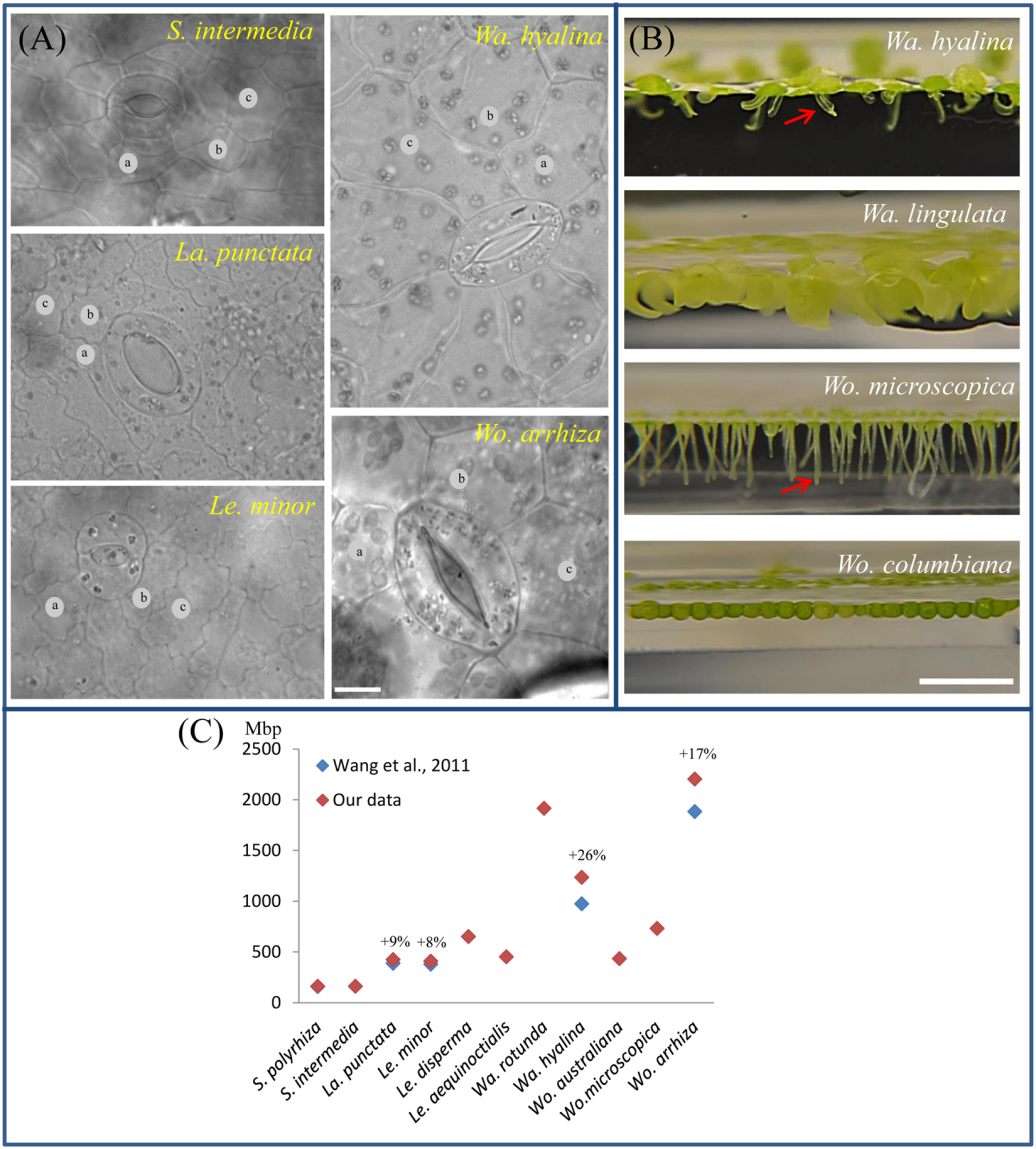
Variation in cell morphology (**A**), floating-style (**B**) and genome size (**C**) in duckweeds. (**A**) Epidermis cell walls are bent in *S*. *intermedia*, undulated in *La*. *punctata*, *Le*. *minor* and rather straight in *Wa*. *hyalina* and *Wo*. *arrhiza*. Stomata are spherical in *S*. *intermedia* and *Le*. *minor*, or elliptic as in *La*. *punctata*, *Wa*. *hyalina* and *Wo*. *arrhiza*. Varying epidermis cell sizes (a–c) in the different duckweed species. (**B**) *Wa*. *hyalina*: Free-floating, two-ovate fronds cohere together. The bent vertical appendage (arrow) is formed from the lower wall of a pouch. *Wa*. *lingulata*: Two tongue-shaped fronds cohere together with frond ends curved downward bringing most of the surface under water. *Wo*. *microscopica*: Free-floating, dorsoventral fronds with irregular polygonal flat dorsal surface and a ventral projection, the pseudo-root (arrow). *Wo*. *columbiana*: Nearly spherical fronds with most of their surface submerged. Stomata are present in the free-floating (*Wa*. *hyalina*, *Wo*. *microscopica*) and almost absent in the submerged (*Wa*. *lingulata*, *Wo*. *columbiana*) species. (**C**) Numbers indicate the deviation of genome size in % (our data relative to that of Wang *et al*.^13^) in the same duckweed clone. Scale bars = 10 µm (**A**), 5 mm (**B**).

The phylogenetic position of the eleven studied duckweed species according to Les *et al*.^16^, the frond morphology and the corresponding genome size is shown in Fig. 1. Both *Spirodela* species have the lowest genome size and the largest fronds, while the genera *Landoltia*, *Lemna*, *Wolffiella* and *Wolffia* have larger genomes (and genome size variation) and progressively smaller fronds. As mentioned by Landolt^9^, duckweed stomata usually stay open and display a slightly higher osmotic value than normal epidermis cells. The open *Spirodela* stomata can close when treated with 3-(4-chlorophenyl)-1 1-dimethylurea, Carbonyl cyanide-4*-(*trifluoromethoxy) phenylhydrazone, valinomycin or nigericin, while these substances had no effect on *Lemna* stomata^9^. Stomata are largely absent in some fully submerged species. Our observation confirmed that not only the frond morphology differs between duckweed genera as described^9^, but also the shape of guard and epidermal pavement cells. Guard cells form spherical stomata in *Spirodela* and *Lemna* species, or elliptic ones as in *Landoltia*, *Wolffiella* and *Wolffia* species (Fig. 1C). Species of the latter two genera show additionally flattened tips of guard cells, compared to the more round ones in *Landoltia*. In all investigated duckweed species displaying stomata, these were usually open. Epidermis cell walls are rather straight in *Wolffiella* and *Wolffia* species, but look bent in *Spirodela* and undulated in *Landoltia* and in *Lemna* species (Fig. 2A). Only very few stomata could be found in *Wa*. *lingulata* and *Wo*. *columbiana*, two largely submerged species (Fig. 2B). To avoid the confusing between *Landoltia* and *Lemna* as well as *Wolffiella* and *Wolffia* genera, we use a two-letter code to abbreviate the names for these genera.

### Genome size variation

The obtained genome sizes varied from 160 Mbp in *S*. *polyrhiza* to 2203 Mbp in *Wo*. *arrhiza* resulting in a ~14-fold difference between duckweed species (Fig. 1). The largest variation in genome size (from 432 to 2203 Mbp) occurred within the genus *Wolffia*. Except for the two *Spirodela* species, our genome size measurements yielded up to 26% larger values than measured for the same clones by Wang *et al*.^13^ (Fig. 2C). In detail, the *S*. *polyrhiza* genome revealed no difference, while a 9% higher value was observed for *La*. *punctata* (7260), 8% for *Le*. *minor* (8623), 17% for *Wo*. *arrhiza* (8872), and 26% for *Wa*. *hyalina* (8640). The differences might be due to different internal reference standards, an unusually low assumption for the genome size of *A*. *thaliana* by Wang *et al*.^13^ (147 Mbp instead of 157 Mbp as measured by Bennett *et al*.^17^) and the use of different flow cytometry equipment.

### Correlation between genome size, nuclear and cell volume within and between duckweed genera

Instead of pavement cells used by Jovtchev *et al*.^14^, we selected guard cells for measurements to investigate a potential correlation between cell parameters of duckweed species with different morphology and genome size. The reason behind is on the one hand the highly variable size and irregular shape of pavement cells (Fig. 2A), that is a challenge for measuring of cell dimensions and for calculating and comparing cell volumes in duckweeds. On the other hand, the permanently open status of stomata in floating aquatic plants^10,18^ yields a rather homogenous guard cell shape, more suitable for precise volume measurement^19^.

**Figure 3:**
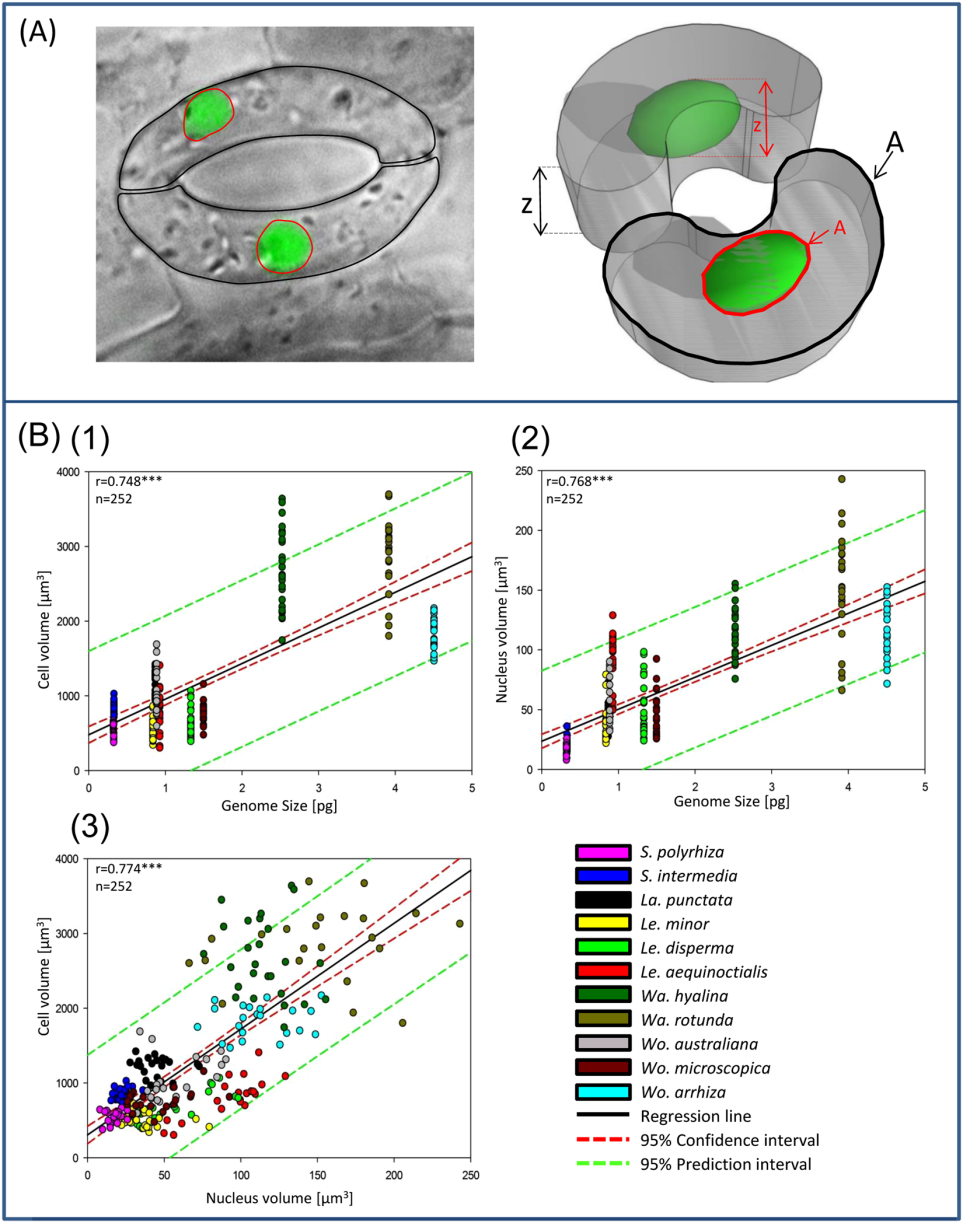
Guard cell and nuclear volume measurement (**A**) and linear regressions of duckweed cell parameters (**B**). (**A**) DIC and fluorescence microscopy image stacks (left) were applied separately (here merged images) to measure the guard cells and the nuclei inside, respectively. The x-y areas (µm^2^) and the z dimension (µm) were measured based on the black (guard cells) and red (nuclei) encircled regions via the ZEN software (spatial illustration, right). (**B**) Regressions between genome size and cell (1) and nucleus volume (2), and between nucleus and cell volume (3). ***p < 0.001 for the correlation coefficient r.

Our results show a moderate but, because of the large number of samples (252) highly significant positive correlation between genome size and cell and nuclear volume in duckweeds. In general, the higher the nuclear DNA content, the bigger are cells and nuclei (Fig. 3 and Table 1). In detail, average cell volume and nuclear volume are 541.7 µm^3^ and 17.1 µm^3^ for *S*. *polyrhiza* (160 Mbp) and increase to 649.6 µm^3^ and 50.3 µm^3^ in *Le*. *disperma* (651 Mbp), and to 1826.8 µm^3^ and 112 µm^3^ in *Wo*. *arrhiza* (2203 Mbp) (Fig. 1B–D). Scatterplots (Fig. 3B) representing all measured data (n = 252) revealed: (i) cell volume and nuclear volume increase with increasing genome size with r = 0.748 and 0.768, respectively; (ii) the cell volume correlates with the nuclear volume (r = 0.774). All correlations are significant at the p < 0.001 level.

**Table 1:** Cytological characterization of eleven duckweeds species.

Genus	*Spirodela*		*Landoltia*	*Lemna*			*Wolffiella*		*Wolffia*		
Species	*polyrhiza*	*intermedia*	*punctata*	*minor*	*disperma*	*aequinoctialis*	*rotunda*	*hyalina*	*australiana*	*microscopica*	*arrhiza*
Clone ID	**7498**	**8410**	**7260**	**8623**	**7269**	**2018**	**9072**	**8640**	**7540**	**2005**	**8872**
Origin	USA	Panama	Australia	Denmark	Australia	Japan	Zimbabwe	Tanzania	New Zealand	India	Hungary
DNA content (pg/2 C)	0.325 ± 0.006	0.327 ± 0.006	0.866 ± 0.012	0.836 ± 0.003	1.331 ± 0.046	0.925 ± 0.003	3.915 ± 0.012	2.523 ± 0.012	0.884 ± 0.012	1.496 ± 0.003	4.505 ± 0.125
Genome size (Mbp/1 C)	160 ± 2	160 ± 3	424 ± 6	409 ± 2	651 ± 3	452 ± 2	1914 ± 6	1234 ± 6	432 ± 6	731 ± 1	2203 ± 61
2n =	40	36	46	42	44	42	82	40	40	40	60
No. 5S rDNA loci	2	2	2	1	1	1	3	2	2	1	3
No. 45S rDNA loci	1	1	1	1	1	1	2	1	1	1	2
Cell volume (µm^3^)	541.7 ± 91.3	855.4 ± 79.1	1204.1 ± 141.3	539.4 ± 130.3	649.6 ± 178.8	812.9 ± 275.8	2859.4 ± 494.5	2665.2 ± 517.6	1087.1 ± 307.9	774.3 ± 134.3	1826.8 ± 216.1
Nuclear volume (µm^3^)	17.1 ± 4.9	22.2 ± 4.5	45.3 ± 11.8	36.4 ± 12.8	50.3 ± 23.3	92.9 ± 21.9	151.9 ± 46.2	115.3 ± 19.5	56.4 ± 19.5	44.7 ± 18.8	111.9 ± 23.3
% nuclear to cell volume	3.2 ± 1.0	2.6 ± 0.5	3.8 ± 1.2	7.3 ± 3.6	7.6 ± 2.1	12.1 ± 2.5	5.5 ± 2.1	4.5 ± 1.3	5.4 ± 1.4	5.8 ± 2.3	6.2 ± 1.4

Error: standard deviation.

Nevertheless, there are exceptions. Within the genus *Lemna*, *Le*. *aequinoctialis* (452 Mbp) showed a larger cell volume (813 µm^3^) and nuclear volume (92.9 µm^3^) than *Le*. *disperma* (651 Mbp, 649.6 µm^3^ and 50.3 µm^3^). A similar result was observed in the genus *Wolffia: Wo*. *australiana* has a smaller genome size (432 Mbp) but a larger cell volume (1087 µm^3^) and nuclear volume (56.4 µm^3^) than *Wo*. *microscopica* (731 Mbp, 774.3 µm^3^ and 44.7 µm^3^) (Fig. 1 and Table 1).

**Figure 4:**
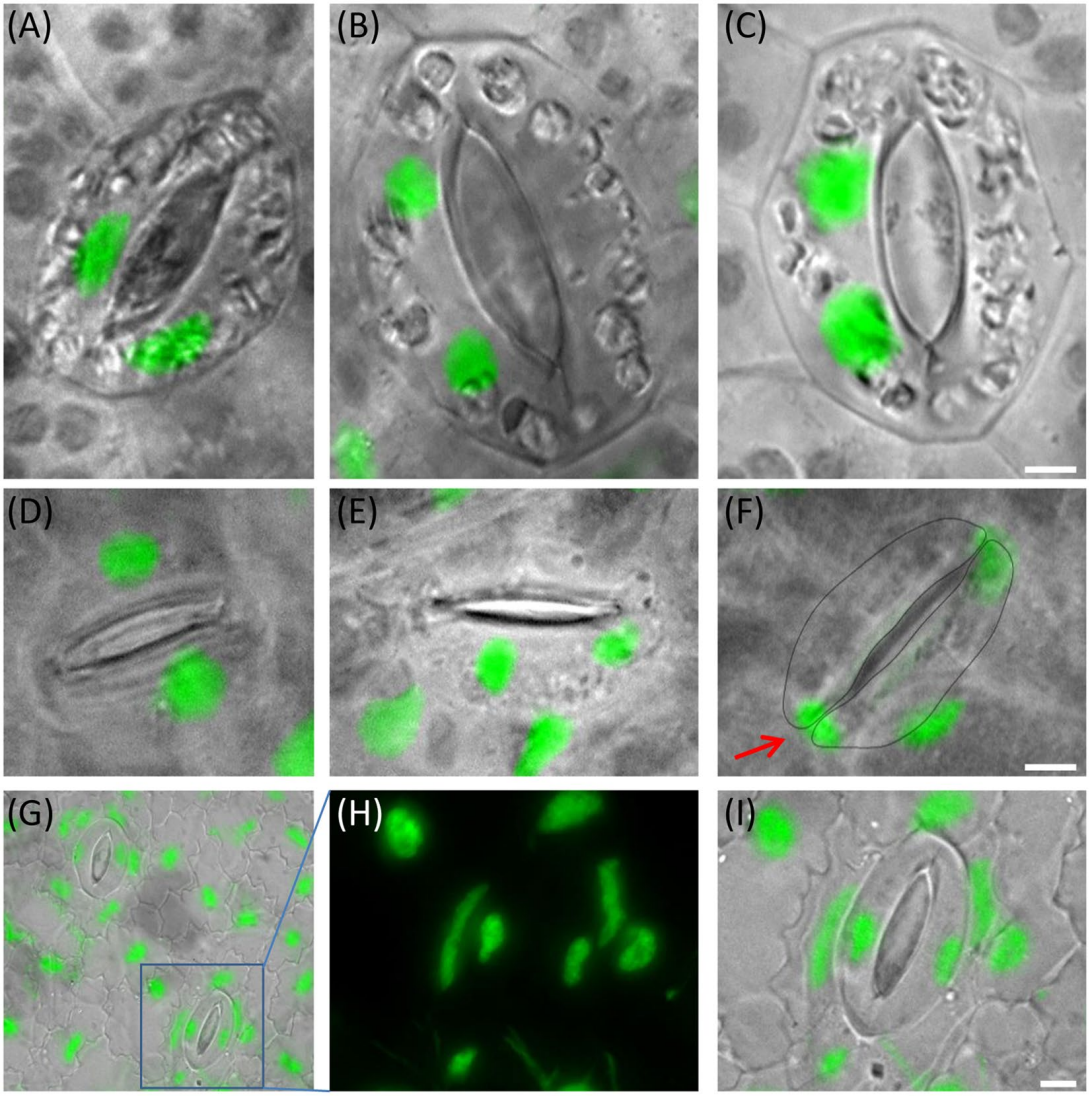
Equal and abnormal nuclei distribution in sister guard cells of *Wa*. *hyalina* (**A**–**C**) and *Wo*. *australiana* (**D**–**F**) and unusual nuclei shape of *La*. *punctata* (5562-A4 mutant) (**G**–**I**). (**A**,**D**) Normal situation (one nucleus per cell); (**B**,**C**,**E**) both nuclei in one sister guard cell; (**F**) the lower nucleus (arrow) is possibly migrating into the sister cell. (**G**) Overview of the nuclei shape in the epidermis of the tetraploid *La*. *punctata* clone 5562_A4 and enlarged frame (**H**,**I**) Scale bars = 5 µm.

Additionally, we found unexpected features in some duckweed species:

(i) *Le*. *aequinoctialis* (2018) revealed a considerable variation in guard cell size and shape (Fig. S1A). In the younger part of frond, guard cells form spherical stomata while in the older part they are elongated and larger. Besides that, cell and nuclear volume are larger than that of *Le*. *disperma* possessing a larger genome. Therefore, we investigated another *Le*. *aequinoctialis* clone (6746) to see whether the variable guard cell volume is specific for this species. Interestingly, this clone showed variation in guard cell size and a nearly doubled genome size (900 Mbp) and correspondingly larger cell and nuclear volumes (1313 µm^3^ and 238 µm^3^, respectively). Thus, the two tested *Le*. *aequinoctialis* clones showed variation not only in guard cell shape, cell volume and nucleus volume, but surprisingly also regarding the genome size (Table S3), most likely due to whole genome duplication (WGD) of clone 6746.
(ii) Both tested *Wolfiella* species, *Wa*. *hyalina* (1234 Mbp, 2665.2 µm^3^ and 115.3 µm^3^) and *Wa*. *rotunda* (1914 Mbp, 2859.4 µm^3^ and 151.9 µm^3^), showed a larger cell and nucleus volume of guard cells than *Wo*. *arrhiza* with a larger genome (2203 Mbp, 1826.8 µm^3^ and 112 µm^3^). Therefore, we wanted to test other *Wolffiella* species to see whether very large cell volume is specific for this genus. Interestingly, only one or two stomata per frond were present in the *Wa*. *lingulata* clone 7725. The same was true for *Wo*. *columbiana* clone 9356. Differences in floating style of *Wo*. *columbiana* with spherical fronds, having most of the surface submerged, and *Wa*. *lingulata* also with a frond shape which keeps most of the frond below the water surface^9^ (Fig. 2B) could be the reason for the almost complete absence of stomata in these species. Thus, so far it remains unclear whether or not a large guard cell size is a typical feature of the genus *Wolffiella*.
(iii) *Wa*. *hyalina* and *Wo*. *australiana* displayed an unusual distribution of nuclei between sister guard cells. We found in 26% of *Wa*. *hyalina* and in 8% of *Wo*. *australiana* guard cells two nuclei located in one sister cell and none in the other (Fig. 4B, C, E). In some cases (6.8% of *Wo*. *australiana* guard cells) it was even possible to find transient stages, suggesting that nuclei may post-mitotically migrate into the sister cell (Fig. 4F). This observation resembles cytomixis, a so far unexplained phenomenon which occurs during microsporogenesis in several higher plants (for review see^20^). These findings, in particular the large variation of guard cell and genome size in *Le*. *aequinoctialis*, and the abnormal nuclei distribution between the sister guard cells are biological features of some duckweeds that deserve further studies.

### Chromosome numbers

Chromosome numbers of duckweed species have been studied by several researchers since 1933 (for references see Tables 2 and S2). However, different chromosome numbers were reported for the same species and it remained unclear whether the discrepancies are due to variation of chromosome number between largely asexual clones within a species. For instance, for different *Le*. *aequinoctialis* clones 40, 50, 66, 72, 78, 84, 65–76 chromosomes^21^, or 40, 50, 60, 80^22^ or only 42 and 84^23^ were counted. For *Wo*. *microscopica*, 70 chromosomes were counted by Roy and Dutt^24^, while Urbanska claimed 40 and 80 chromosomes^22^.

**Table 2:** Chromosome numbers of 11 tested duckweed species from our study and others.

Genus	Species	Clones	2n	Source	Genus	Species	Clones	2n	Source
***Spirodela***	***polyrhiza***	7652	30	U	***Lemna***	***disperma***	7818	40	U
		7621	40				7223, 7190	44	W
		7110	50				7269*	**44**	**O**
		8118, 7205, 7120, 7160, 7687, 8483, 8403, 8409, 6613, 7003, 7206, 6731, **7498**, 8442, 8229, 7212, 7551, 7674, 7960, 7222, 7379, 6581	40	W		***aequinoctialis***	7382	20	U
							8038	40	
							7204	50	
							8079	60	
		**7652**, **7657**, 7364	30				6746	80	
		7110	80	G			6612	40	W
		6613, 7667, 7364, 7551, S7, S3	40				7126	60	
							6746	80	
		**7498**, **7652**, **7657**	**40**	**O**			6746	84	G
	***intermedia***	7747	20	U			2018*	**42**	**O**
		7201	30				**6746**	**~80**	
		**8410**, 7355, 8258, 7747, 8818, 7178	36	G	***Wolffiella***	***hyalina***	7426	**40**	U
							7378, 7376, **8640**	40	W
		**8410**	**36**	**O**			**8640**	**40**	**O**
***Landoltia***	***punctata***	8028	40	U		***rotunda***	9072*	**82**	**O**
		7479	50		***Wolffia***	***australiana***	7819	20	U
		**7449**, 7248	40	W			**7540**	40	
		**7260**	50				7733	20	W
		O5, O6, 7461, 7191, 7799, 7429	46	G			**7540**	**40**	**O**
						***microscopica***	7238	40	U
		**7260**, **7449**	**46**	**O**			8359	80	
***Lemna***	***minor***	7798	20	U			M8	70	R
		7244	30				2005*	**40**	**O**
		6626	40			***arrhiza***	7251	30	U
		7572	42				**8272**	40	
		6742	50				7193	50	
		**8623**, 7018, 7210, 8434, 7436, 7136	40	W			7699	60	
							7158	70	
		7123, 6591	42	W			7196	80	
		7189, 8676, 7789, 7244	42	G			7347	42	G
		M4, 7114, 7182, 8653	63	G			**8872**	**60**	**O**
		7115	126	G					
		**8623**	**42**	**O**					

(R) Roy & Dutt^24^; (U) Urbanska^22^, (G) Geber^23^, (W) Wang *et al*.^13^, (O) our study

Bold and underlined: clones were used in our study; (*) clones were counted for the first time.

Among 34 *S*. *polyrhiza* clones mentioned by Wang *et al*.^13^, the chromosome number of nine clones was not determined, for three clones (7652, 7657 and 7364) 2n = 30, and for the other clones 2n = 40 was reported (Table 2).

**Figure 5:**
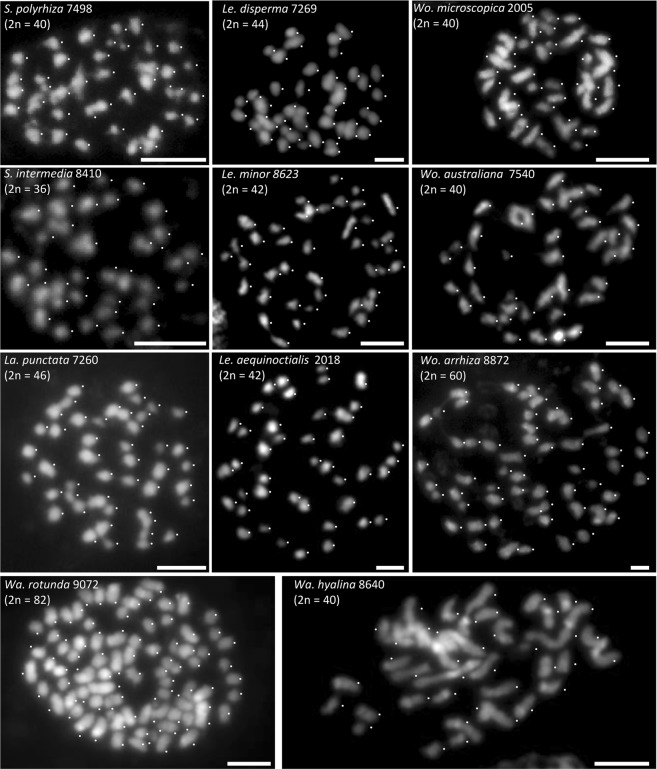
Chromosome numbers of eleven duckweed species, identified in somatic metaphases. Scale bars = 5 µm.

Here, we selected clones 7652 and 7657 for chromosome counting and found 2n = 40, as in clone 7498 (Figs. 5 and S2) and in further six *S*. *polyrhiza* clones^25^. For *S*. *intermedia*, 2n = 36 was reported by Geber^23^ in all six tested clones, while Urbanska^22^ counted 2n = 20 (clone 7747) and 2n = 30 (clone 7201) (Table 2). Here, we selected *S*. *intermedia* clones 8410 and 7747 for chromosome counting and found 2n = 36 for both clones (Figs 5 and S2). Similarly, for *La*. *punctata*, we counted 2n = 46 for clones 7260, 5562 and 7449 (Fig. S2), while 50 and 40 chromosomes were reported for clones 7260 and 7449, respectively^13^ (Table 2). Therefore, in all investigated clones of *S*. *polyrhiza* (7498, 7652, 7657, 9500, 9505, 9507, 9509, 9510 and 9511), *S*. *intermedia* (8410 and 7747) and *La*. *punctata* (5562, 7260 and 7449), no variation of chromosome number was observed.

Our chromosome counting results are mainly similar to that of Geber^23^ (Table 2). In detail, *S*. *polyrhiza* showed 2n = 40, *S*. *intermedia* 2n = 36, *La*. *punctata* 2n = 46, *Le*. *disperma* 2n = 44, and *Le*. *minor* 2n = 42. For *Wo*. *australiana* (clone 7540) we counted 2n = 40 as reported by Urbanska^22^, for *Le*. *aequinoctialis* (clone 2018) 2n = 42, and for *Wa*. *hyalina* 2n = 40. For *Le*. *disperma* (clone 7269, 2n = 44), for *Le*. *aequinoctialis* (clone 2018, 2n = 42), for *Wo*. *microscopica* (clone 2005, 2n = 40) and for *Wa*. *rotunda* (clone 9072, 2n = 82), chromosomes were counted for the first time in our study. Meanwhile *Wo*. *microscopica* clones used by Urbanska^22^ and by Roy and Dutt^24^ got lost and therefore cannot be re-investigated. In case of *Wo*. *arrhiza*, 42 chromosomes were counted by Geber^23^ (clone 7347), or 30, 40, 50, 60, 70 and 80 chromosomes for different clones by Urbanska^22^ while we counted 60 chromosomes for clone 8872 (Table 2).

### Intraspecific variation of genome size, chromosome number and guard cell parameters

Different chromosome numbers were found in different clones of *Le*. *aequinoctialis*^23^, 42 chromosomes were counted for clones 7382, 7321, 7300 and 7737, while 84 chromosomes were counted for clones 6746 and 7384. Meanwhile, these clones (except 6746) were lost from international duckweed collections. We chose the *Le*. *aequinoctialis* clone 2018 instead for ploidy testing within this species. As described above, genome size varies from 452 Mbp (clone 2018) to 900 Mbp (clone 6746). These data suggest that clone 6746 is tetraploid. We investigated the correlation between genome size, cell and nuclear volume and counted chromosome number of the two *Le*. *aequinoctialis* clones (6746 and 2018). In parallel, two clones of *La*. *punctata:* clone 7260 (diploid) and clone 5562_A4 (a true artificial tetraploid) were included.

Both genome size measurement and chromosome counting suggest that *Le*. *aequinoctialis* clone 6746 is tetraploid with larger cell and nuclear volume, and clone 2018 is diploid with smaller cell and nuclear volume (Table S3). Fig. S1B represents all measured data (n = 40, p < 0.001) and revealed a positive correlation between cell and nuclear volume (r = 0.593).

A similar result was obtained for the two clones of *La*. *punctata* clones 7260 and 5562_A4 (Table S3 and Fig. S1C). In addition, the tetraploid *La*. *punctata* clone 5562_A4 frequently showed elongated instead of round nuclei (Fig. 4G–I). Cell and nucleus volumes are significantly different (at least at p = 0.01 level) for diploid and tetraploid clones of both species. Therefore, the 95% confidence intervals do not overlap (Fig. S1B,C).

### Location of 5S and 45S rDNA loci on duckweed chromosomes

A remarkably low copy number of 45S rDNA (18S and 26S rDNA) but also of 5S rDNA was reported for *S*. *polyrhiza*^25^. A significant decrease in copy number of 45S rDNA has apparently occurred in *S*. *polyrhiza* (81 copies) compared to the 13-times smaller genome of *Saccharomyces cerevisiae* (~12.2 Mbp/1 C) with 150 copies^26^, or the similar-sized genome of *Arabidopsis thaliana* with 570 copies^27^. The locus of 45S rDNA is located on chromosome ChrS 01 and two loci of 5S rDNA on ChrS 13 and ChrS 06 with 60 and 12 copies, respectively^25,28^.

**Figure 6:**
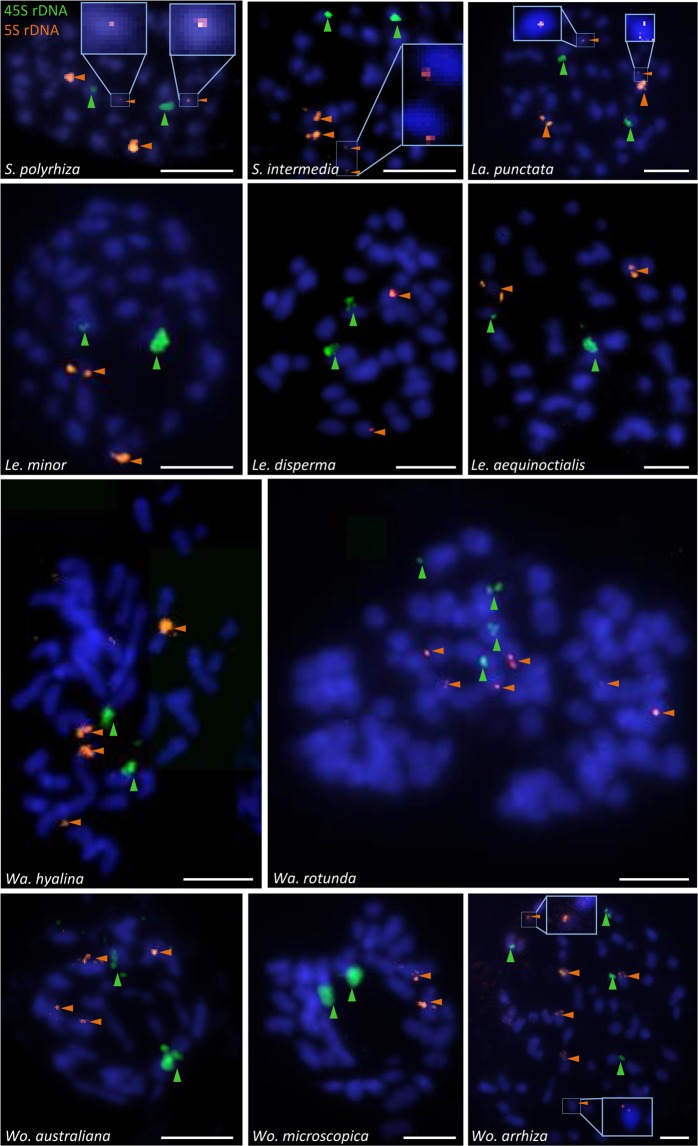
5S and 45S rDNA loci (arrowheads) of eleven duckweed species. Two loci of 5S and one locus of 45S rDNA were detected on *S*. *polyrhiza*, *S*. *intermedia*, *La*. *punctata*, *Wa*. *hyalina*, *Wo*. *australiana*; one locus of 5S and 45S each were detected on *Le*. *minor*, *Le*. *disperma*, *Le*. *aequinoctialis* and *Wo*. *microscopica;* three loci of 5S and two loci of 45S rDNA were detected in *Wa*. *rotunda* and *Wo*. *arrhiza*. Framed: minor loci of 5S rDNA. Scale bars = 5µm

The number of 45S and 5S rDNA loci of the eleven studied duckweed species was determined by FISH (Table 1, Fig. 6). In detail, one locus of 45S and 5S rDNA each was detected in *Le*. *minor*, *Le*. *disperma*, *Le*. *aequinoctialis*, *Wo*. *microscopica*, while *S*. *polyrhiza*, *S*. *intermedia*, *La*. *punctata*, *Wa*. *hyalina* and *Wo*. *australiana* displayed one locus of 45S rDNA and two loci of 5S rDNA. In *Wo*. *arrhiza*, two loci of 45S rDNA and three loci of 5S rDNA were detected.

In *Wa*. *rotunda* (clone 9072), three loci of 5S rDNA were detected and two chromosome pairs displayed 45S rDNA loci. One pair of NORs was more extended and showed a distal satellite (Figs. 6 and S3B). Without rDNA FISH signals, the satellite distal to the NOR could erroneously be counted as a small pair of chromosomes. The strength of FISH signals reflected differences in copy number of 5S rDNA. For instance, the 5S rDNA probe often yielded in *Wo*. *arrhiza* (clone 8872) two strong, two medium and two weak FISH signals. Noticeably, a very low copy number of 5S rDNA could apparently prevent the detection by FISH, e.g. the 5S rDNA locus with only 12 copies on ChrS 06 of *S*. *polyrhiza*^25^. Weak signals of 5S rDNA loci (in *S*. *polyrhiza*, *S*. *intermedia*, *La*. *punctata* and *Wo*. *arrhiza*) could only be detected in a few metaphases (Fig. 6), and thus are at risk to be overlooked. Therefore, the number of 5S rDNA loci which were detected by FISH in other duckweed species than *S*. *polyrhiza* might underestimate the true number of loci as long as their genomes are not completely assembled.

## Discussion

Our measurements of genome size in relation to frond and cell shapes, guard cell volume, nuclear volume, chromosome number and number of rDNA loci for eleven species, representative for the five duckweed genera, led to several conclusions or speculations, or pointed to further open questions:

(i) Some duckweed species seem to have specific frond and cell structures which are suitable for different floating-styles (totally, largely or not submerged) and are not strongly affected by genome size.
(ii) Genome size is known to correlate with a number of traits in angiosperms. DNA content and nuclear volume as well as nuclear and cell volume showed positive correlation at different endopolyploidy levels in epidermis cells of *A*. *thaliana* (from 2C to 32C), *Barbarea stricta* (from 2C to 16C) as well as between species that differ in genome size up to ~500 fold (from 0.32 pg in *A*. *thaliana* to 154.99 pg in *Fritillaria uva-vulpis*)^14^ or between 14 herbaceous angiosperm species^29^. A correlation of cell parameters (DNA content, cell volume, nuclear volume, cell surface, nucleus surface) was also reported for *Sorghum bicolor* endosperm cells from 3C to 96C^15^. In this study, cell and nuclear volumes from guard cells of the eleven duckweeds species provided in total a significant positive correlation between genome size, nuclear and cell volume. However, this correlation is not as strong as for cells of different endopolyploidy levels within one species^14,15^. The weaker correlation is likely caused by the fact that individual duckweed species may have an own specific body and cell structure and size, and a range of intraspecific variation of these features which might blur the influence of genome size on nuclear and cell volume.
(iii) Genome size differences between duckweed species rise the question to what degree frond size and neoteny level are correlated with the genome size, which was previously shown not to be correlated with an organisms’ complexity^30,31^. In general, genome size (and genome size variation) increases with the reduced morphological differentiation in duckweeds. However, there are some exceptions: In spite of similar genome sizes of about 400 Mbp, frond size and neoteny level differ between *La*. *punctata*, *Le*. *minor* and *Wo*. *australiana*, while species, with similar neoteny level, may own different genome size, e.g. *Le*. *minor* (409 Mbp), *Le*. *disperma* (651 Mbp). The genome size variation between *Le*. *aequinoctialis* clones 2018 and 6746 (452 and 900 Mbp) might be due to WDG, because also the chromosome number is doubled in clone 6746, and is accompanied by larger nuclear and cell volumes (Fig. S1B). Whether the large genome size differences between duckweed genera as well as between species within the genera *Lemna*, *Wolffiella* and especially *Wolffia* are based on WGD or on a retroelement burst remains to be solved. It might also be that DNA double-strand break repair biased towards deletions or duplications^32,33^ plays a role in genome size variation, e.g. between *Wolffia* species. It also remains unclear why at all genome size increases with decreasing organismic complexity and decreasing frond size of duckweeds and whether or not this correlation results in a lower (and possibly constant) cell number.
(iv) Mitotic chromosome spreads of all tested species (Fig. 5) revealed that, as expected, genome size is not correlated with chromosome number. That means, genome size and chromosome number vary independently from each other.
(v) No chromosome number variation was detected between the tested clones of *Spirodela* and *Landoltia* species. The reported high variation of chromosome number in the phylogenetically younger genera *Lemna*, *Wolffia* and *Wolffiella* (as summarized in Fig. 1, Tables 2 and S2) needs further investigation to be confirmed or disproved. In case of confirmation it will be of interest to elucidate the mechanisms behind.
(vi) Ribosomal genes (rDNA) are characterized by conserved sequences and organized as tandem repeat units in eukaryotic genomes. Variations regarding number and chromosomal distributions of 5S and 45S rDNA loci are informative markers for discriminating karyotypes of species, and in specific cases, for elucidating karyotype evolution, for instance in Brassicaceae^34,35^ and in Anthemideae^36^. In the eleven tested duckweed species, the observed number of 5S and 45S rDNA loci revealed no correlation with chromosome number and/or genome size. Whether the extremely low copy number of rDNA sequences, as observed for *S*. *polyrhiza*, is typical for duckweeds has to be checked when complete sequences of further duckweed genomes will be available. Completely sequenced genomes will also reveal whether FISH experiments detected all 5S rDNA loci so far, or whether additional minor loci escaped from detection as was the case for the locus on chromosome 6 of *S*. *polyrhiza* with only 12 copies^25^.

## Materials and Methods

### Plant material and mitotic chromosome preparation

*S*. *polyrhiza* (accession 7498) and *S*. *intermedia* (8410) were obtained from Elias Landolt via BIOLEX (Pittsboro, NC, USA) and Rutgers Duckweed Stock Cooperative (New Jersey, USA) (Table 1). *S*. *polyrhiza* (7652 and 7657), *S*. *intermedia* (7747), *La*. *punctata* (7260, 7449), *Le*. *minor* (8623), *Le*. *disperma* (7269), *Le*. *aequinoctialis* (2018, 6746), *Wa*. *hyalina* (8640), *Wa*. *rotunda* (9072), *Wo*. *microscopica* (2005), *Wo*. *australiana* (7540) and *Wo*. *arrhiza* (8872) were from K.-J. Appenroth’s collection. These eleven species have been chosen because they cover the ranges of genome size variability between and within genera, are of different geographic origin and were available in the collections. *La*. *punctata* 5562 and its colchicine-induced tetraploid mutant 5562_A4 were obtained from M. Edelman, Rehovot, Israel. The fronds were grown in liquid nutrient medium^37^ under 16 h white light of 100 µmol m^−2^ s^−1^ at 24 °C.

Spreading of mitotic chromosomes was carried out according to Cao *et al*.^38^ with some modifications. In brief, healthy fronds were incubated in 2 mM 8-hydroxyquinoline at 37 °C and then fixed in fresh 3:1 absolute ethanol: acetic acid for at least 24 h. The samples were washed twice in 10 mM Na-citrate buffer, pH 4.6, for 10 min each before and after softening in 2 ml pectinase/cellulase enzyme mixture, prior to maceration and squashing in 60% acetic acid. After freezing on dry ice or in liquid nitrogen, the slides were treated with pepsin, post-fixed in 4% formaldehyde in 2xSSC (300 mM Na-citrate, 30 mM NaCl, pH 7.0) for 10 min, rinsed twice in 2xSSC, 5 min each, dehydrated in an ethanol series (70, 90 and 96%, 2 min each) and air-dried (Table S1).

### Genome size measurement

Genome size measurements were performed according to Dolezel *et al*.^11^ using a CyFlow Space flow cytometer (Sysmex/Partec). For nuclei isolation and staining the DNA staining, kit ‘CyStain® PI Absolute P’ was used. As internal reference standards either *Raphanus sativus* ‘Voran’ (IPK gene bank accession number RA 34; 2C = 1.11 pg) for *S*. *polyrhiza*, *S*. *intermedia*, tetraploid *La*. *punctata*, *Le*. *minor*, *Wa*. *hyalina*, *Wo*. *australiana*, *Wo*. *microscopica*, *Glycine max* (L.) Merr. convar. *max* var. *max*, Cina 5202 (IPK gene bank accession number SOJA 32; 2C = 2.21 pg) for *La*. *punctata*, *Wa*. *rotunda*, *Le*. *disperma* *or Lycopersicon esculentum* Mill. convar. *infiniens* Lehm. var. *flammatum* Lehm., Stupicke Rane (IPK gene bank accession number LYC 418; 2C = 1.96 pg) for *Le*. *aequinoctialis*, *Wo*. *arrhiza* were used. The absolute DNA contents (pg/2C) were calculated based on the values of the G1 peak means and the corresponding genome sizes (Mbp/1C) according to Dolezel *et al*.^39^. In total, for each species at least six independent measurements on two different days were performed.

### Epidermis preparation, microscopic cell and nuclear volume measurements, and statistics

Due to the small frond size, a single epidermis layer is difficult to obtain especially for species of the genus *Wolffia* (frond diameter ~1 mm). Therefore, we modified the epidermis preparation methods described^40–42^, by using domestic adhesive tape. Because stomata are located on the upper surface in floating plants^9,18^, duckweed fronds were placed with their upper side on the adhesive tape. Other parts of the fronds were carefully removed with a razor blade until only the transparent layer of epidermis stuck on the tape. Ten µl of DAPI (2 µg/ml) in Vectashield were dropped on slides before the adhesive tape with the epidermis layer was placed on slides and covered by a coverslip. Freshly prepared slides were used immediately to avoid the disintegration of the nuclei before imaging. Differential interference contrast (DIC) and fluorescence (excitation of DAPI with a 405 nm laser) image stacks were acquired using a Super-resolution Fluorescence Microscope Elyra PS.1 and the software ZEN (Carl Zeiss GmbH). The DIC image stacks were used to measure the x-y area A, and the z dimension of the guard cells via the ZEN software. Accordingly, the image stacks were used to measure the nuclei dimensions (Fig. 3A). These dimensions were applied to calculate the guard cell and nuclear volumes by the following formulae:

$${\rm{Cell}}\,{\rm{Volume}}={{\rm{A}}}_{{\rm{cell}}}\,\ast \,{\rm{z}}$$

$${\rm{Nuclear}}\,{\rm{volume}}=2/3\,\ast \,{{\rm{A}}}_{{\rm{nucleus}}}\,\ast \,{\rm{z}}$$

It means, the guard cells are considered as stacks with the base area A and the height z, while the nuclei are considered as ellipsoids.

The correlation values (Pearson product moment correlation coefficient) and the corresponding p values were calculated with the program SigmaPlot 12 (Systat Software, Inc.). The same program was used for the plot of the regression diagrams. At least 20 sister guard cells (ten stomata) with the corresponding nuclei were chosen for measurements per species.

### Fluorescence ***in situ*** hybridization (FISH) and microscopy

Genomic DNA of *S*. *polyrhiza*, *La*. *punctata*, *Le*. *minor*, *Wa*. *hyalina and Wo*. *arrhiza* were used as template to amplify rDNA regions with designed primer pairs for:

(i) 18S-rDNA: 18S–SSU1(F) (TGGTTGATCCTGCCAGTAG) and 18S–1243 R: (AGAGCTCTCAATCTGTCA)^43^;
(ii) 26S-rDNA: 26S–0091 F (TAGTAACGGCGAGCGAACC)^2^ and 26S–1229rev (ACTTCCATGACCACCGTCCT)^44^;
(iii) 5S rDNA: UP46 (GTGCGATCATACCAGCACTAATGCACCGG) and UP47 (GAGGTGCAACACGAGGACTTCCCAGGAGG)^45^.

Telomere-specific probes were generated by PCR using tetramers of the Arabidopsis-type telomere repeats without template DNA according to Ijdo *et al*.^46^. PCR products were used as templates for PCR-labeling (5S rDNA) or nick-translation (18S, 26S rDNA and telomere sequences) to generate the corresponding FISH probes. The probes were labeled with Cy3-dUTP (GE Healthcare Life Science), Alexa Fluor 488-5-dUTP, Texas Red-12-dUTP, biotin-dUTP or digoxigenin-dUTP (Life Technologies) and precipitated as described in Hoang and Schubert^47^.

Probes were denatured at 95 °C for 5 min and chilled on ice for 10 min before adding 10 µl probe per slide (up to three different labeled probes simultaneously). Then, the mitotic chromosome preparations were denatured together with the probes on a heating plate at 80 °C for 3 min, followed by incubation in a moist chamber at 37 °C for at least 16 h. Post-hybridization washing and signal detection were carried out according to Lysak *et al*.^48^. Widefield fluorescence microscopy for signal detection followed Cao *et al*.^38^. The images were pseudo-colored and merged using Adobe Photoshop software (ver.12)  (Adobe Systems).

To analyze the ultrastructure and spatial arrangement of signals and chromatin at a lateral resolution of ~120 nm (super-resolution, achieved with a 488 nm laser), 3D structured illumination microscopy (3D-SIM) was applied using a Plan-Apochromat 63x/1.4 oil objective of an Elyra PS.1 microscope system and the software ZENblack (Carl Zeiss GmbH). Image stacks were captured separately for each fluorochrome using the 561, 488, and 405 nm laser lines for excitation and appropriate emission filters^49^. Maximum intensity projections of whole cells were calculated via the ZEN software. Zoom in sections were presented as single slices to indicate the subnuclear chromatin structures at the super-resolution level.

## Supplementary information

Supplementary Dataset 01
