# Supplementary material for: Variation in genome size, cell and nucleus volume, chromosome number and rDNA loci among duckweeds
Source: Sci Rep. 2019 Mar 1;9:3234. doi: 10.1038/s41598-019-39332-w (PMC6397220; doi:10.1038/s41598-019-39332-w)
Supplement: Supplementary file 1 — Supplementary Dataset 01 [file 41598_2019_39332_MOESM1_ESM.pdf]

Supplementary figures and tables:

Variation in genome size, cell and nucleus volume, chromosome number and rDNA loci among duckweeds

Phuong TN Hoang[1,2], Veit Schubert[1], Armin Meister[1], Jörg Fuchs[1], Ingo Schubert[1*]

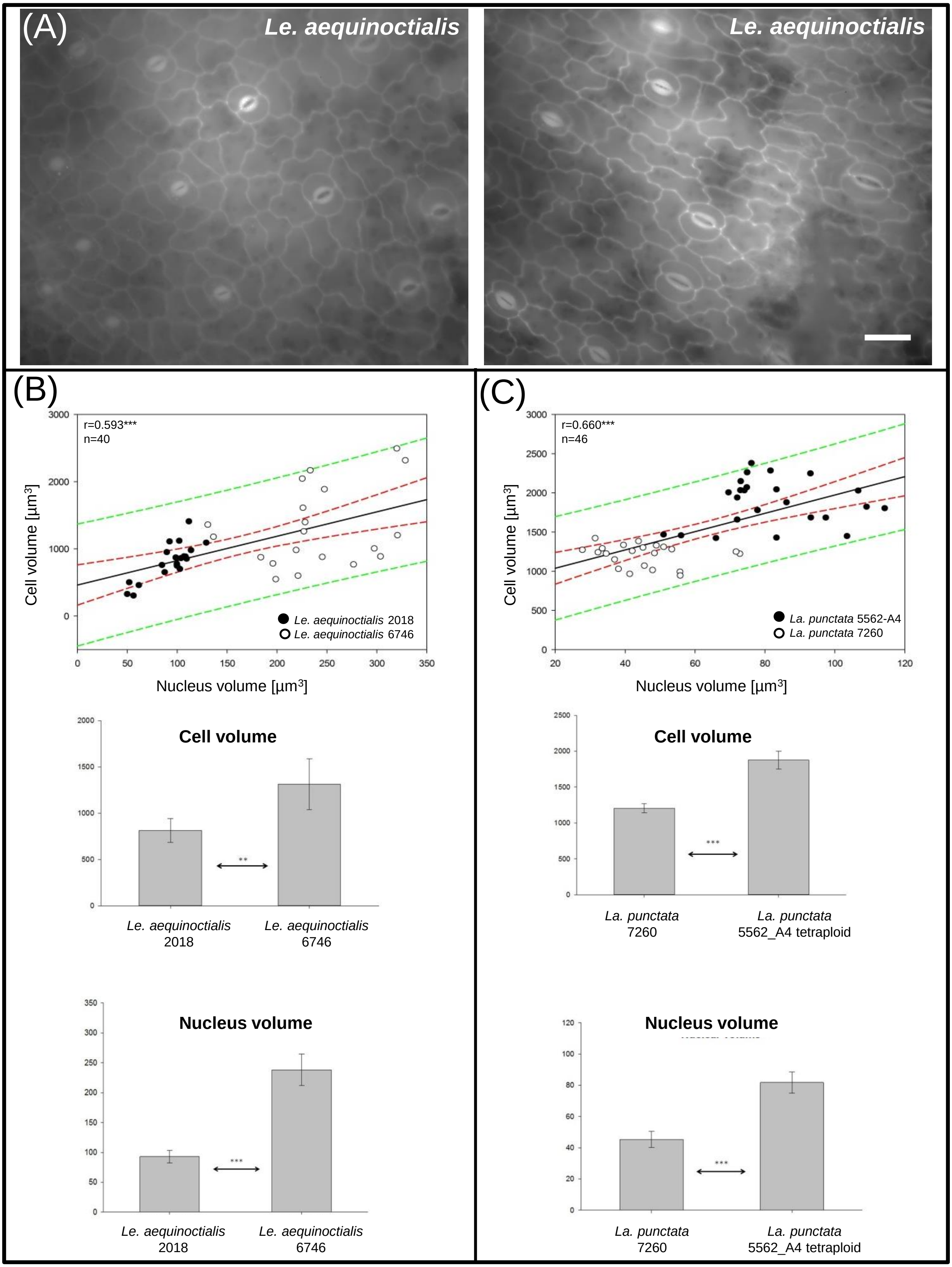

FIGURE S1: Variation in guard cell shape and volume of Le. aequinoctialis (A) and correlation of cell parameters in clones of *Le. aequinoctialis* (B) *and La. punctata* (C) which differ two-fold in genome size. (A) Guard cells of spherical appearance in a younger region (left) and elongated ones in an older region (right) are of different size (a,b,c) in *Le. aequinoctialis* (clone 2018); (B, C) Correlations between cell and nucleus volume (top) and variation of cell (middle) and nucleus (bottom) volume between cells of different genome size. **$p<0.01$, *** $p < 0.001$; regression line (black); 95% confidence interval (red) and 95% prediction interval (green). Bar charts: Error bars: 95% confidence interval; double arrows: result of mean value comparison by t-test.

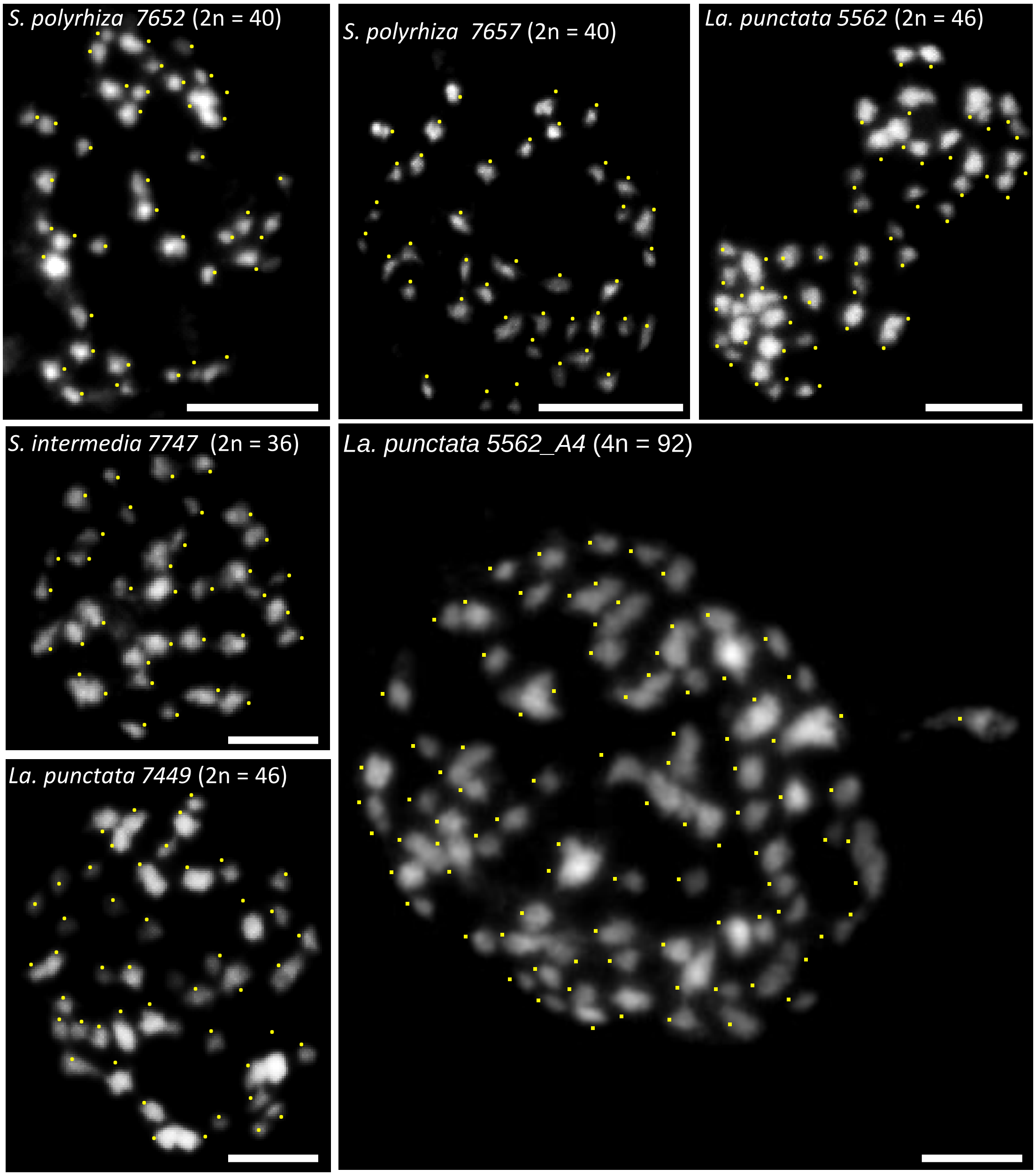

FIGURE S2: Chromosome numbers counted in mitotic metaphases of *S. polyrhiza, S. intermedia* and *La. punctata* clones.
Scale bar = 5 µm

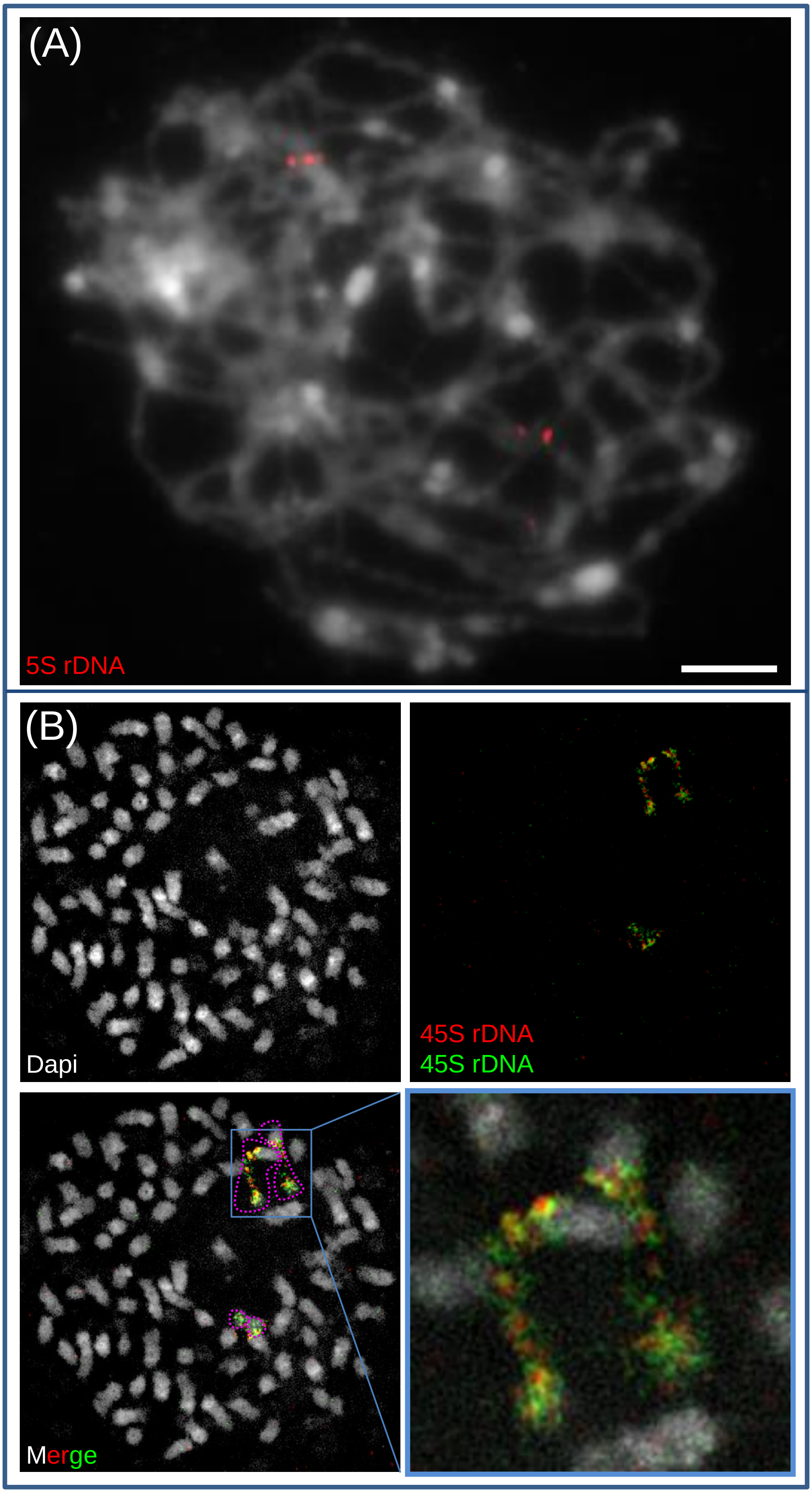

FIGURE S3: rDNA FISH signals in pachytene (A) and mitotic metaphase (B) of *Wa. rotunda* (clone 9072) using super-resolution microscopy (SIM).
(A) Three loci of 5S rDNA on pachytene chromosomes; (B) 45S rDNA signals (red and green) on a metaphase spread label two chromosome pairs (pink dots, bottom left). The enlarged frame (bottom right) shows the upper pair with extended secondary constrictions. Without the FISH signals, the satellites distal the NOR could erroneously be counted as small chromosome pair. Scale bar = 5 µm

Table S1: Chromosome preparation procedures for duckweed species

Species	Tissue	Metaphase arrest (*)	Cell wall digestion (**)		Protein digestion (***)	Slide freezing
			Enzyme concentration	Time		
S. polyrhiza	Meristem	3.5 h	1.0 %	60 min	7 min	Dry ice (30 min or more)
S. intermedia	Meristem	3.5 h	1.0 %	60 min	7 min	Dry ice (30 min or more)
La. punctata	Meristem	2.5 h	0.5 %	30 min	5 min	Dry ice (30 min or more)
Le. minor	Meristem	1.5 h	0.5 %	15 min	5 min	Liquid nitrogen (5 min)
Le. disperma	Meristem	1.5 h	0.4 %	10 min	5 min	Liquid nitrogen (5 min)
Le. aequinoctialis	Meristem	1.5 h	0.4 %	8 min	3 min	Liquid nitrogen (5 min)
Wa. hyalina	Meristem	2.0 h	0.4 %	8 min	5 min	Liquid nitrogen (5 min)
Wa. rotunda	Meristem	2.0 h	0.4 %	8 min	15 min	Liquid nitrogen (5 min)
Wo. microscopica	Whole frond	2.0 h	0.4 %	8 min	3 min	Liquid nitrogen (5 min)
Wo. australiana	Whole frond	2.0 h	0.4 %	10 min	3 min	Liquid nitrogen (5 min)
Wo. arrhiza	Whole frond	2.0 h	0.4 %	12 min	3 min	Liquid nitrogen (5 min)

(*) 2 mM 8-hydroxylquinoline at 37°C
(**) Cellulase and pectinase mixture in sodium citrate buffer, pH 4.6 at 37°C
(***) 50 µg/ml pepsin in 0.01N HCl at 37°C

Table S2: Chromosome number of duckweed species were counted from literatures

Genus	Species	2n	Source
**Spirodela**	***polyrhiza***	40	1*
		40	2*
		32, 40	3*
		30, 40	13
		30, 40, 50	11
		40,8	12
	intermedia	20, 30	11
		36	12
**Landoltia**	***punctata***	40, 43-44, 50	11
		40, 50	13
		46	12
**Lemna**	***aequinoctialis***	20, 40, 50, 60, 80	11
		42, 84	12
		40, 60, 80	13
		40, 50, 66, 72, 78, 84, 65-76	4*
	disperma	40	11
		44	12
	gibba	64	1*
		ca. 60	2*
		40, 50, 70, 80	11
		42, 43, 44, 84-86	12
	japonica	40, 50	11
		50	13
		63	12
	minuscula	36, 40	11
		42	12
	minor	40	1 *
		42	5*
		40	6*
		40	2*
		50	7*
		20, 30, 40, 42, 50	11
		40, 42	13
		42, 63, 126	12
	obscura	40, 50	11
		42	12
	perpusilla	40	11
		42	12
	turionifera	40, 42, 50, 80	11
		42	12

Genus	Species	2n	Source
**Lemna**	***trisulca***	44	1*
		Ca. 40	2*
		20, 40, 60, 80	11
		40, 60, 80	13
		42, 44, 63-66	12
	valdiviana	40	11
		42	12
**Wolffiella**	***denticulata***	20, 40	11
	gladiata	42	8*
		40	13
		40	11
	hyalina	40	13
		40	11
	lingulata	42	8*
		20, 40, 50	13
		20, 40, 50	11
	neotropica	40	11
	oblonga	42	8*
		40, 70	11
		42	12
	welwitschii	40	11
**Wolffia**	***arrhiza***	ca. 50	1*
		44-46	9*
		50	2*
		62	**
		30, 40, 50, 60, 70, 80	11
		42	12
	australiana	20	13
		20, 40	11
	angusta	40	11, 13
	borealis	20, 30, 40	11
	brasiliensis	42	**
		20, 40, 50, 60, 80	11
	columbiana	Ca. 42	8*
		40	13
		30, 40, 50, 70	11
	globosa	30, 40, 50, 60	11
		60	13
		46	**
	microscopica	70	10
		40, 80	11

(1) Blackburn (1933); (2) Wcislo (1970); (3) Banerjee (1971); (4) Beppu&Takimoto (1981); (5) Brooks (1940); (6) Delay (1947); (7) Loeve (1978); (8) Daubs (1965); (9) Lawalree (1943); (10) Roy &Dutt[24]; (11) Urbanska[22]; (12) Geber[23]; (13) Wang et al.[13], *: mentioned in Geber[23]; **: Kwanyumen (personal communication) mentioned in Urbanska[22].

Table S3: Cell parameters of *La. punctata* and *Le. aequinoctialis* clones of two-fold genome size difference

Genus	Landoltia		Lemna	
Species	*punctata*		*aequinoctialis*	
Clone ID	**7260**	**5562_A4***	**2018**	**6746**
Origin	Australia	Israel	Japan	USA
DNA content (pg/2C)	0.866± 0.012	1.885 ± 0.007	0.925 ± 0.003	1.841 ± 0.008
Genome size (Mbp/1C)	424 ± 6	922 ± 4	452 ± 2	900 ± 4
2n	46	92	42	~80
Cell volume (μm^3)	1204.1 ± 141.3	1905.1 ± 290.3	812.9 ± 275.8	1313.3 ± 588.3
Nuclear volume (μm^3)	45.3 ± 11.8	80.2 ± 15.9	92.9 ± 21.9	238.2 ± 56.4
% Nuclear to cell volume	3.8 ± 1.2	4.3 ± 0.9	12.1 ± 2.5	21.3 ± 9.5

± : Standard deviation; *colchicine-induced tetraploid;
